# Resistance-induced brain activity changes during cycle ergometer exercises

**DOI:** 10.1186/s13102-021-00252-w

**Published:** 2021-03-19

**Authors:** Ming-An Lin, Ling-Fu Meng, Yuan Ouyang, Hsiao-Lung Chan, Ya-Ju Chang, Szi-Wen Chen, Jiunn-Woei Liaw

**Affiliations:** 1 Faculty of Computer and Software Engineering, Huaiyin Institute of Technology, Huaian, Jiang-Su China; 2 Department of Occupational Therapy and Graduate Institute of Behavioral Science, School of Medicine, Chang Gung University, Taoyuan, Taiwan; 3 Division of Occupational Therapy, Department of Rehabilitation, Chiayi Chang Gung Memorial Hospital, Chiayi, Taiwan; 4 Department of Electrical Engineering, Chang Gung University, Taoyuan, Taiwan; 5 Department of Neurology, Chang Gung Memorial Hospital, Linkou, Taiwan; 6 Neuroscience Research Center, Chang Gung Memorial Hospital, Linkou, Taiwan; 7 School of Physical Therapy and Graduate Institute of Rehabilitation Science, College of Medicine, and Health Aging Research Center, Chang Gung University, Taoyuan, Taiwan; 8 Department of Electronic Engineering, Chang Gung University, Taoyuan, Taiwan; 9 Department of Mechanical Engineering, Chang Gung University, Taoyuan, Taiwan; 10 Center for Advanced Molecular Imaging and Translation, Chang Gung Memorial Hospital, Linkou, Taiwan

**Keywords:** Cycle ergometer exercise, Exercise intensity, Electroencephalogram, Phase-locking value, Brain connectivity

## Abstract

**Background:**

EEGs are frequently employed to measure cerebral activations during physical exercise or in response to specific physical tasks. However, few studies have attempted to understand how exercise-state brain activity is modulated by exercise intensity.

**Methods:**

Ten healthy subjects were recruited for sustained cycle ergometer exercises at low and high resistance, performed on two separate days a week apart. Exercise-state EEG spectral power and phase-locking values (PLV) are analyzed to assess brain activity modulated by exercise intensity.

**Results:**

The high-resistance exercise produced significant changes in beta-band PLV from early to late pedal stages for electrode pairs F3-Cz, P3-Pz, and P3-P4, and in alpha-band PLV for P3-P4, as well as the significant change rate in alpha-band power for electrodes C3 and P3. On the contrary, the evidence for changes in brain activity during the low-resistance exercise was not found.

**Conclusion:**

These results show that the cortical activation and cortico-cortical coupling are enhanced to take on more workload, maintaining high-resistance pedaling at the required speed, during the late stage of the exercise period.

## Background

Physical exercise is known as an efficient means to reduce anxiety [1], improve motor function [2–6] and even enhance cognitive performance [7, 8]. The improvement of cerebral function through exercise has been linked to significant changes in various electroencephalogram (EEG) metrics, such as rest-state power [1, 9, 10], asymmetry [11], and event-related potentials [2, 8].

An alternative way to describe brain activity is in terms EEG coherence or phase synchronization, the degree of co-modulation between two cerebral regions. Rest-state EEG coherence measurements have been used to detect changes in cortico-cortical couplings after (but not during) short-term exercise. For example, studies have found increased coherence between sensorimotor areas after graded cycle ergometer exercise [6], increased fronto-temporal and cortico-cerebellar coherences after a dynamic pincer-grasp task [12], and even a reorganization of cortico-cortical couplings after combined cognitive and physical training in patients with mild cognitive impairment [13].

In addition to rest-state EEGs, exercise-state EEGs are analyzed to assess brain activity modulated by exercise or a motor task. An increase in EEG power is associated with exercise intensity [14], and also with muscular fatigue during various types of exercise: cycle ergometer [15, 16] and sustained isometric hand-grip contraction [17]. Exercise-state EEG coherence or phase synchronization is used as an index to study cerebral adaption during fatigue. EEG synchronization increases as the mental state of drivers shifts from alertness to fatigue [18, 19], and shed light on the reorganization of cortico-cortical couplings during mental fatigue [20, 21] or driving fatigue [22]. Increased cortico-cortical coupling has also been observed during the fatigue periods of elbow extension contraction [23] and cycling exercise [24]. In addition, brain network connectivity was modulated during an endurance cycling exercise [25]. The aforementioned exercise-induced change in cortico-cortical coupling or network connectivity is demonstrated during the sustained constant-intensity exercise [23] or the incremental graded-intensity exercise [24, 25] which may derive from the demands that exercise and motor work make on cognitive processing.

In literatures, EEG coherence and phase synchronization as well as EEG power in alpha and beta bands are mostly used to study brain connectivity [23–25] and brain activation [10, 15] during exercise. The beta rhythm is regarded as an important role in motor control, whereas the modulation of alpha rhythm is associated with different stages of exercise [26]. Higher frequency EEG such as the gamma rhythm has been shown to be involved in sensory processing, movement control, memory and attention but it is challenging to analyze this rhythm because of the spectral overlapping with muscle activity [27].

Exercise intensity is usually adjusted depending on the subject’s status, or to achieve a desired training effect. The specific effect of exercise intensity on brain activity has mostly been studied with respect to changes in EEG power. One study used incremental graded cycling exercises [10, 15], and another measured EEG power during handgrip tasks with different maximal voluntary contractions separated by 10-min rests [28]. Note that these variable-intensity exercises were conducted on the same day. They therefore do not exclude the effect of cumulative fatigue confounded between different intensity conditions.

This study aims to understand the impact of exercise intensity on brain activity using a separate-day experimental design, in order to exclude the effects of cumulative fatigue that may be present in previous studies of this type. That is, we conduct experiments of different exercise intensities on different days. Moreover, although there is ample evidence that inter-brain cooperative processing should also be modulated by exercise intensity, to date no controlled exercise intensity experiments have measured EEG coherences or phase synchronizations. Hence, the purpose of this study is also to investigate the response of cerebral collaboration (EEG phase-locking values) to low- and high-resistance cycle ergometer exercise. The experiments are conducted by ten healthy subjects, each subject performing high-intensity and low-intensity exercise on two separate days a week apart. Multi-channel EEGs are recorded during the exercises.

## Methods

### Participants

Ten healthy, right-handed, male subjects (age 21.40 ± 1.02 y, height 171.90 ± 4.68 cm, weight 64.20 ± 5.15 kg) were chosen for cycle ergometer exercises (C016–1702, Body Sculpture Inc., Taiwan). The sample exclusion criteria were: 1) skeletal muscular disease; 2) neuromuscular disease; 3) use of anti-depressant drugs; and 4) participating in lower limb strengthening exercise within a week before the study. The tension of the flywheel can be magnetically controlled from level 1 to level 8. Each participant was asked to sit in an upright position and perform low-resistance (level 4) or high-resistance (level 8) exercise on two different days a week apart. The order of exercise intensity was counterbalanced across subjects. Half the subjects performed the low-intensity exercise first, and the other half performed the high-intensity exercise first. The participants were randomly assigned an exercise order.

### Cycle ergometer exercises

**Fig 1:**
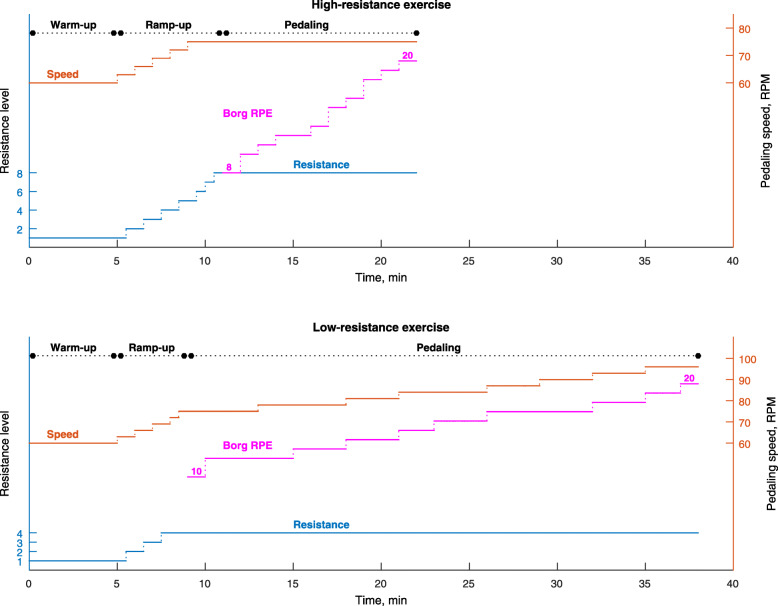
Timeline of adjustments in cycling speed and resistance level over the course of two experiments in an exemplary subject: one with high resistance (top) and one with low resistance (bottom). The left-hand portions of the plots show the initial warm-up and ramp-up phases. The Borg rating of perceived exertion (RPE), in purple, shows the value reported by the subject during the sustained pedaling phase after ramp-up

Each participant was instructed to perform warm-up pedaling to prevent exercise injury. For the first 5 min, the flywheel tension was set to level 1 and the subject maintained a pedaling speed of 60 RPM (revolutions per minute), guided by a metronome. After the warm-up period, the subjects entered a ramp-up period where the pedaling speed and flywheel tension were increased alternately every 30 s. The pedaling speed increased by 3 RPM at each adjustment until 75 RPM was reached; the flywheel tension increased by one level until the target resistance (4 or 8) was reached. According to the cycle ergometer’s calibration, the target powers for the low- and high-resistance exercises were 70 and 140 W respectively. The left-hand portions of the curves in Fig. 1 show the adjustments in cycling speed and flywheel tension for the low- and high-resistance exercises in an exemplary subject. For the low-resistance exercise, it took 9 min to reach the target resistance and speed. For the high-resistance exercise, it took 11 min to reach the targets.

After the warm-up and ramp-up phases, each participant performed a sustained pedal exercise at an initial speed of 75 RPM. A rating of perceived exertion (RPE) on the Borg scale was obtained every minute by asking the participant how hard he felt the exercise was. The Borg scale ranges from 6 to 20, with 6 representing no exertion at all and 20 representing maximal exertion [29]. (The subject described intermediate values as extremely light, very light, light, somewhat hard, hard, very hard, or extremely hard.) The Borg RPE has been widely used as a subjective estimate of exercise effort and intensity [30–32]. Simultaneous increase of perceived exertion, heart rate and blood lactate as the Borg RPE was observed during exercise on a bicycle ergometer [32, 33]. A study showed if a subject reported the same RPE for a 3 min period, we asked him to increase his pedaling speed by 3 RPM. The pedaling exercise stopped when the RPE scale reached 20 or when the participant had pedaled for 30 min. This protocol is used to avoid exercise injury because the subject felt extremely hard or took too much time in pedaling. The right-hand portions of the curves in Fig. 1 show the Borg RPE values and the adjusted cycling speed during the low- and high-resistance exercises in the exemplary subject. This subject reported the same RPE at 12 during the 10th – 12th min and at 17 during the 26th – 28th min because of less effort required to cope low-resistance pealing. Accordingly, the pedal speed was asked to increase from 75 to 78 RPM at the 13th min and from 90 to 93 RPM at the 29th min, respectively. After speed adjustment, he reported the same RPE for 2 and 3 min respectively.

### Data collection and analysis

The EEGs were measured through an EEG cap with 32 silver/silver chloride coated electrodes which are wired to the EEG recording system (SynAmps RT, Compumedics Neuroscan, Victoria, Australia). These scalp electrodes were placed according to the international 10/20 system: F3, C3, P3, O1, F4, C4, P4, O2, F7, T3, T5, F8, T4, T6, Fz, Cz, Pz, Oz and vertical electrooculogram. The electrodes are referenced to the average mastoids with impedances less than 5 KΩ before exercise. EEG activities are amplified with a gain of 1000 and digitised with a sampling rate of 1000 Hz. The raw digital EEG signals are detrended using a 4th–order, anti-causal, Butterworth highpass filter with a cutoff frequency of 0.5 Hz and filtered by a notch filter at 60 Hz to eliminate powerline interference.

Independent vector analysis is used to decompose multi-channels EEGs into an equal number of source components [34]. The ocular artefact-related components are identified and discarded based on the criteria of greater delta-band power and gradient decreasing from anterior to posterior brain regions [35]. Since muscle artefacts is expected to be present in the lowest autocorrelated sources [36, 37], the muscle artefact-related components are identified based on a normalized autocorrelation index (NAI). The NAI is defined as the mean of autocorrelations for lags 0.6 to 2 s divided by the autocorrelation at zero lag. The source component with the least NAI is excluded. The remaining source components are therefore used to reconstruct EEG signals.

The reconstructed EEGs are emphasized by a 4th–order anti-causal Butterworth highpass filter with a cutoff frequency at 3 Hz to remove low-frequency movement artifacts including cycling-inducing motion artifacts. The instantaneous EEG intensity is given by the channel-wise root mean square of the emphasized EEGs over F3, C3, P3, F4, C4, P4, Fz, Cz, and Pz used to detect abruptly changed components in the emphasized EEGs. The emphasized EEGs are also filtered by a 4th–order anti-causal Butterworth lowpass filter with a cutoff frequency at 300 Hz used to compute EEG power and phase-locking values.

The sustained pedal exercise (after warm-up and ramp-up are complete) is divided into three equal-duration stages (early, middle and late). In each stage, we select the first 50 s of the filtered EEG signal for data analysis. Each 50 s EEG sample *x*(*t*) is divided into 99 overlapping 1-s segments with a Blackman window (50% window overlap). The first 75 segments satisfying that all instantaneous intensity within the segment is less than 125 μV are selected for subsequent analysis. The selected segments are analyzed separately by a discrete Fourier transform with a spectral bin width of 1 Hz. The auto spectral density is the magnitude squared of the transform. The averaged auto spectral density *P*_*av*_*xx*(*f*) is obtained by averaging the individual auto spectral densities *Pxx*(*k*,*f*) of the selected 1-s segments (*M* = 75):

$$ {P}_{av} xx(f)=\frac{1}{M}\sum \limits_{k=1}^M Pxx\left(k,f\right) $$

Two parameters are computed from the averaged auto spectral density. The alpha-band power (α) is defined as the total power spectral density between 8 and 13 Hz, and the beta-band power (β) is the total between 14 and 20 Hz.

Phase-locking value (PLV) is a statistic that examines the linear relationship between two signals used to quantify the functional association between two brain areas. The PLV can separate the phase and amplitude components, so it is less affected by synchronous fluctuations in power [38]. Two 4th–order, anti-causal, Butterworth bandpass filters are used to decompose the EEG signals into two narrow-band signals *s*(*t*) corresponding to alpha (8–13 Hz) and beta rhythms (14–20 Hz). These decomposed signals are then transformed to their analytic form using the Hilbert transform:

$$ z(t)=s(t)+ jHT\left\{s(t)\right\} $$

The relative phasor between two narrow-band analytic signals, *z*_x_(*t*) and *z*_*y*_(*t*) is calculated as

$$ {e}^{j\Delta  \varphi (t)}=\frac{z_x(t){z}_y^{\ast }(t)}{\left|{z}_x(t)\right|\left|{z}_y(t)\right|} $$

The alpha-band PLV is defined as the absolute value of the average of the relative phasors obtained from the alpha-band analytic signals in the above-selected segments. The beta-band PLV is derived in the same way. Brain connectivity is assessed as the alpha- and beta-band PLVs obtained from two kinds of associations: intra-hemispheric links (F3–C3, C3–P3, Fz–Cz, Cz–Pz, F4–C4, C4–P4, F3-Fz, Fz-F4, C3-Cz, Cz-C4, P3-Pz, Pz-P4) and inter-hemispheric links (F3–F4, C3–C4, P3–P4).

### Statistical analysis

The α power, β power, PLV_α_, and PLV_β_ are treated as dependent variables to assess brain activity. The Borg RPE and mean heart rate are used to quantify the feeling of fatigue and the autonomic control of the heart during exercise [39, 40]. The number of variables for each type is a multiplicity of 6 from the three stages and two exercise intensities, and another multiplicity from the nine electrodes or fifteen electrode pairs. We assess the normality of all variables using the Shapiro–Wilk test, obtaining 21 out of 54 for α power, 42 out of 54 for β power, 86 out of 90 for PLV_α_, 80 out of 90 for PLV_β_, 4 out of 6 for Borg RPE, and 6 out of 6 for the mean heart rate that passed the normality test (*p* > 0.05). Since the sample size is limited, and since the assumptions of normality and heterogeneity of variance are violated for some variables, we used nonparametric statistics to measure the difference in two distributions of a dependent variable, such as an EEG power measured for low-intensity exercise in early-stage or late-stage pedaling for the 10 subjects.

The paired permutation test is used to make nonparametric, pairwise comparisons of a dependent variable measured in the early, middle, and late pedal stages (*p* < 0.05/3) for the same subject. In addition, the effects of exercise resistance on brain activity is tested by calculating the change in a dependent variable from early to middle stages (middle - early) or from early to late stages (late - early) and compare its value between the low- and high-resistance exercises using the paired permutation test (*p* < 0.05).

The effect size is reported as the rank correlation coefficient ρ which is approximated as the z score divided by the square root of the total number of scores based on the Wilcoxon signed-rank test. The strength of the effect can be considered low if the absolute value of the correlation coefficient is around 0.1, medium if it is around 0.3, and large if it is more than 0.5 [41, 42].

## Results

### Effects of exercise on Borg RPE and mean heart rate

In this study, an exercise session ceased when the Borg RPE reached 20 or the pedal time (not including warm-up and ramp-up) exceeded 30 min. The duration for the low-resistance pedaling (median = 1569.0 s, interquartile range (iqr) =772.6 s) was significantly higher than so did the high-resistance pedaling (median = 459.9 s, iqr =308.2 s) with a low *p* value (*p* = 0.0028). Table 1 lists the Borg RPE and mean heart rate measured in the early, middle and late pedal stages. Both metrics increased significantly from early to middle to late stages in both exercises. This result demonstrates that the feeling of fatigue and the sympathetic activity really do increase as the exercise progresses, so the early, middle, and late pedal stages are physiologically meaningful.

**Table 1:** Borg RPE and mean heart rate in various pedal stages during the low- and high-resistance exercises

	Early	Middle	Late
Low-resistance			
*Borg RPE*	9 (1)	12.5 (2)^**†**^	16 (2)^**†‡**^
*Mean heart rate*	106.7 (13.9)	111.8 (12.7)^**†**^	119.4 (14.8)^**†‡**^
High-resistance			
*Borg RPE*	13 (0)	16 (2)^**†**^	18 (1)^**†‡**^
*Mean heart rate*	137.8 (23.5)	150.8 (20.8)^**†**^	157.6 (22.3)^**†‡**^

All data are represented as median (interquartile range). The paired permutation test is used to compare the differences between two pedal stages. The mark † indicates that the middle or late stage variable is significantly different from its early-stage value (p < 0.05/3). The mark ‡ indicates that the late-stage variable is significantly different from its middle-stage value (***p*** **< 0.05/3).**

In addition, a low *p* value and a high effect size (ρ) were shown in Borg RPE among pedal stages in low-resistance exercise (middle vs. early: *p* = 0.0014, ρ = 0.629; late vs. early: *p* = 0.0020, ρ = 0.633; late vs. middle: *p* = 0.0026, ρ = 0.631) and high-resistance exercise (middle vs. early: *p* = 0.0024, ρ = 0.643; late vs. early: *p* = 0.0012, ρ = 0.637; late vs. middle: *p* = 0.0016, ρ = 0.643). Mean heart rate also exhibited a low *p* value and a high effect size among pedal stages in low-resistance exercise (middle vs. early: *p* = 0.0023, ρ = 0.627; late vs. early: *p* = 0.0022, ρ = 0.627; late vs. middle: *p* = 0.0037, ρ = 0.604) and high-resistance exercise (middle vs. early: *p* = 0.0018, ρ = 0.627; late vs. early: *p* = 0.0021, ρ = 0.627; late vs. middle: *p* = 0.0034, ρ = 0.597).

### Brain activities in different pedal stages

The number of 1-s EEG segments that contain abruptly changed components in early, middle, and late pedal stages is 1.50 ± 1.84, 1.40 ± 3.13, and 0.70 ± 1.64 during low-resistance exercise; 5.10 ± 7.40, 2.70 ± 5.81, and 4.90 ± 8.40 during high-resistance exercise. There was neither statistical significance between low- and high-resistance pedal stages nor statistical significance among early, middle and late stages. In order to make all stages have the same number of the analyzed segments, we include the first 75 segments without abruptly changed components for spectral and PLV analysis.

**Fig. 2:**
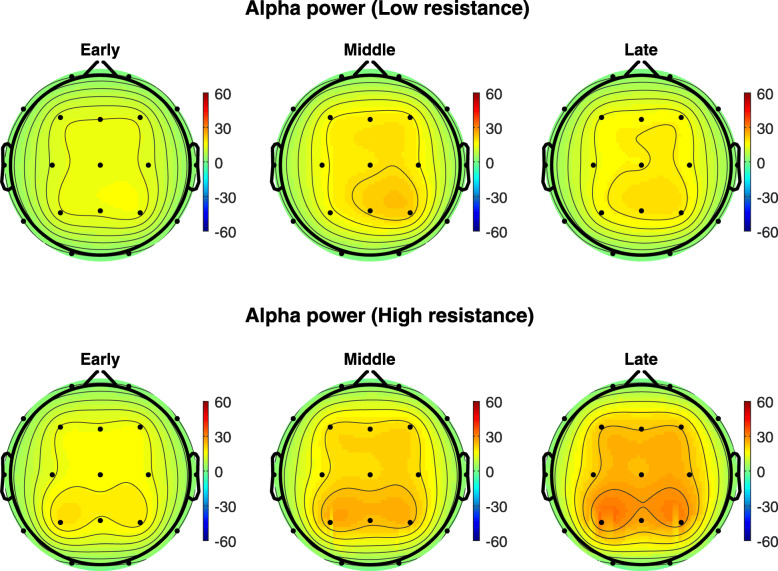
The topographical maps of the grand average of alpha-band powers over all subjects in μV^2^ across 9 electrodes (F3, Fz, F4, C3 Cz, C4, P3, Pz, P4) during the low- and high-resistance pedal exercises

**Fig. 3:**
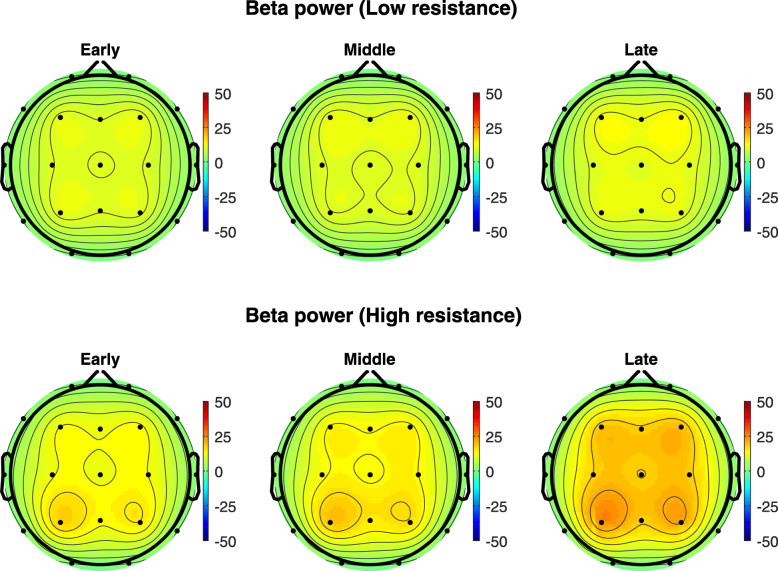
The topographical maps of the grand average of beta-band powers over all subjects in μV^2^ across 9 electrodes (F3, Fz, F4, C3 Cz, C4, P3, Pz, P4) during the low- and high-resistance pedal exercises

Figure 2 shows the topographical maps of the grand average of alpha-band powers, measured during the low- and high-resistance pedal exercises, over all subjects across 9 electrodes (F3, Fz, F4, C3 Cz, C4, P3, Pz, P4); Fig. 3 the band-band power. Higher spectral power is exhibited during high-resistance, late pedal stage, in particular in the parietal area.

**Fig. 4:**
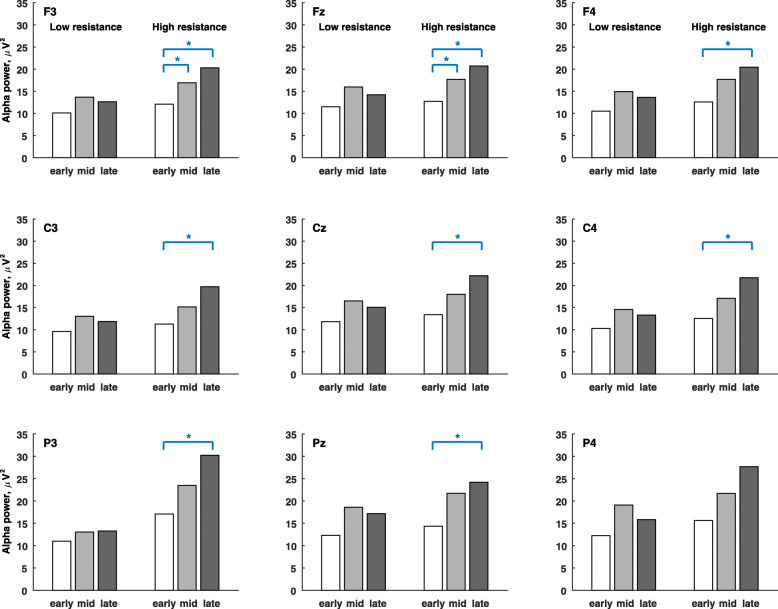
The grand average of alpha-band powers during early, middle and late pedal stages in μV^2^. The paired permutation test is used to compare the differences between pedal stages. The mark * indicates that the variable is significantly different between pedal stages (*p* < 0.05/3)

**Fig. 5:**
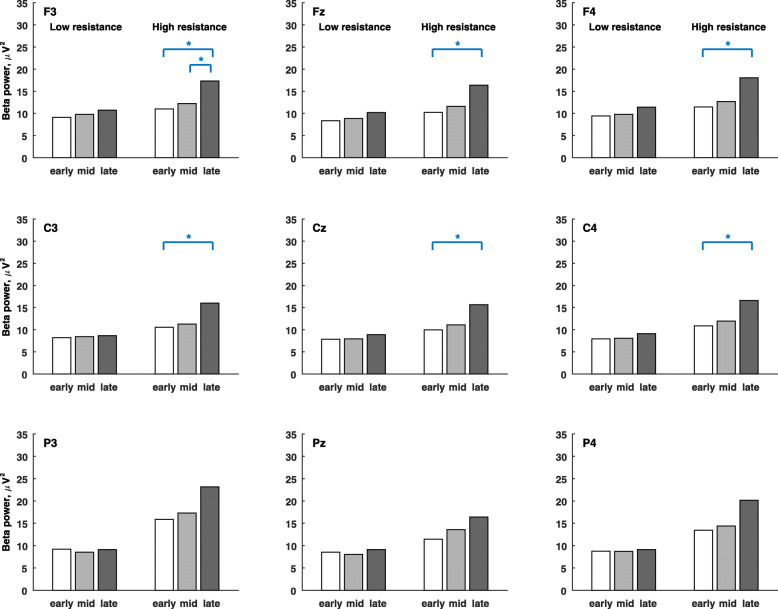
The grand average of beta-band powers during early, middle and late pedal stages in μV^2^. The paired permutation test is used to compare the differences between pedal stages. The mark * indicates that the variable is significantly different between pedal stages (*p* < 0.05/3)

Figure 4 shows the grand average of alpha-band powers in the low- and high-resistance pedal stages. The alpha power was significantly higher in the late stage than the early stage at electrodes F3, Fz, F4, C3 Cz, C4, P3, and Pz, and also significantly higher in the middle stage than the early stage at electrodes F3 and Fz during the high-resistance exercises. Figure 5 shows the grand average of beta-band powers in the low- and high-resistance pedal stages. The beta power was significantly higher in the late stage than the early stage at electrodes F3, Fz, F4, C3 Cz, and C4, and also significantly higher in the late stage than the middle stage at electrode F3 during the high-resistance exercise.

**Fig. 6:**
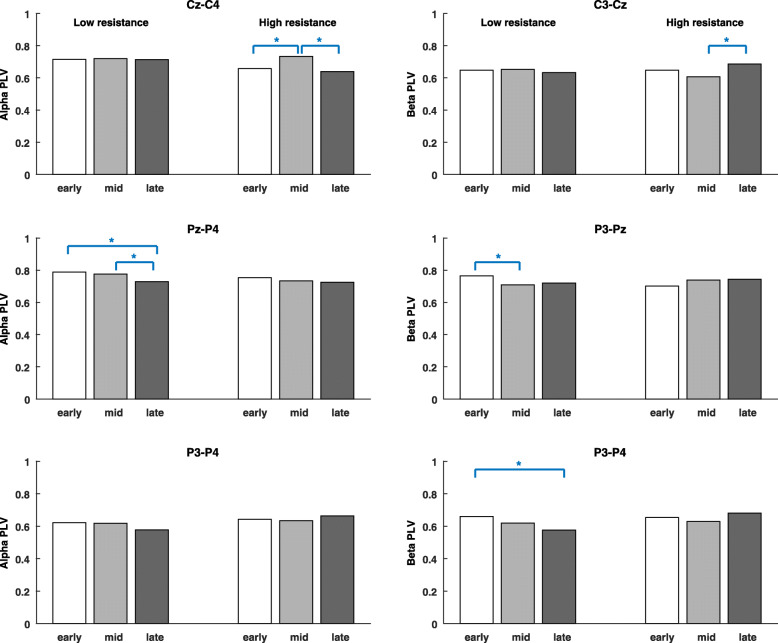
The grand average of alpha- and beta-band phase-locking values during early, middle and late pedal stages. The paired permutation test is used to compare the differences between pedal stages. The mark * indicates that the variable is significantly different between pedal stages (*p* < 0.05/3)

Figure 6 shows the alpha- and beta-band phase-locking values (PLV_α_ and PLV_β_) during early, middle and late pedal stages. For low-resistance exercise, PLV_α_ in the Pz-P4 link significantly decreased from early to middle to late stages, and PLV_β_ in the P3-Pz and P3-P4 link was individually lower in the middle or late stage than in the early pedal stage. In contrast, PLV_α_ in the Cz-C4 link was the highest in the middle stage, and PLV_β_ in the C3-Cz link was significantly higher in the late stage than in the middle stage, for high-resistance exercises.

### Changes of brain activities across exercise resistances

Change of brain activity is quantified by the change rate of alpha-band power (Δα) and beta-band power (Δβ) from early to middle and from early to late pedal stages, during both low- and high-resistance exercises. For example, Δα from early to middle stages is defined as (α_middle_ - α_early_) / α_early_.

Table 2 lists the change rates of alpha- and beta-band power (Δα and Δβ) from early to middle and from early to late pedal stages. We find that the Δα values from early to late stages, measured at electrodes C3 and P3, were significantly higher during high-resistance pedaling than low-resistance pedaling.

**Table 2:** Change rate of alpha-band power (Δ α) and beta-band power (Δ β) from early to middle and from early to late pedal stages

Inter-cerebral pairs	Δ α		Δ β	
	Middle - Early	Late - Early	Middle - Early	Late - Early
C3				
*Low-intensity*	0.344 (0.836)	0.162 (0.456)	−0.027 (0.656)	0.035 (0.557)
*High-intensity*	0.259 (0.616)	0.466 (0.173)	0.019 (0.378)	0.415 (1.257)
*p-value*	0.5028	0.0113*	0.7041	0.1332
*z-score*	−0.561	2.395	0.663	1.478
*Effect size, ρ*	−0.125	0.536	0.148	0.331
P3				
*Low-intensity*	0.244 (0.677)	0.115 (0.494)	−0.077 (0.479)	−0.038 (0.484)
*High-intensity*	0.124 (1.328)	0.422 (0.451)	−0.1006 (0.333)	0.345 (0.775)
*p-value*	0.4667	0.0224*	0.5409	0.2442
*z-score*	0.663	2.293	0.357	1.172
*Effect size, ρ*	0.148	0.513	0.080	0.262

Δα and Δβ are the change rates in the alpha-band power (α) and beta-band power (β) between pedal stages. For example, Δα from early to middle stages is defined as (α_middle_ - α_early_) / α_early_. The change rate is represented as median (interquartile range). The paired permutation test is used to compare the distributions of change rates between low- and high-resistance exercises, where the mark * indicates a significance level of *p* < 0.05. The effect size is reported as the correlation coefficient ρ based on the Wilcoxon signed-rank test

Table 3 lists the changes in inter-cerebral alpha- and beta-band phase-locking values (ΔPLV_α_ and ΔPLV_β_) from early to middle and from early to late pedal stages during low- and high-resistance exercises. Significantly larger PLV changes from early to late stages were observed during high-resistance pedaling than low-resistance pedaling.

**Table 3:** Changes of inter-cerebral phase-locking values (ΔPLV _α_ and ΔPLV _β_) from early to middle and from early to late pedal stages

Inter-cerebral pairs	ΔPLV _α_		Δ PLV _β_	
	Middle - Early	Late - Early	Middle - Early	Late - Early
C3-Cz				
*Low-intensity*	0.044 (0.186)	0.012 (0.247)	0.026 (0.208)	0.033 (0.140)
*High-intensity*	0.023 (0.073)	0.025 (0.170)	−0.034 (0.036)	0.052 (0.111)
*p-value*	0.5004	0.8544	0.2616	0.0333*
*z-score*	0.765	0.051	−1.070	2.090
*Effect size, ρ*	0.171	0.011	−0.239	0.467
P3-Pz				
*Low-intensity*	0.017 (0.092)	0.005 (0.112)	−0.028 (0.087)	−0.026 (0.086)
*High-intensity*	−0.028 (0.131)	0.002 (0.068)	0.004 (0.162)	0.040 (0.155)
*p-value*	0.7840	0.4312	0.0091*	0.0412*
*z-score*	−0.051	0.561	2.497	1.886
*Effect size, ρ*	−0.011	0.125	0.558	0.422
P3-P4				
*Low-intensity*	−0.012 (0.148)	−0.029 (0.121)	− 0.065 (0.097)	−0.082 (0.084)
*High-intensity*	0.037 (0.259)	0.054 (0.250)	−0.054 (0.149)	0.019 (0.127)
*p-value*	0.9261	0.0165*	0.7766	0.0147*
*z-score*	−0.255	2.293	0.153	2.293
*Effect size, ρ*	−0.057	0.513	0.034	0.513

PLV changes (Middle - Early, Late - Early) are represented as median (interquartile range). ΔPLV_α_ and ΔPLV_β_ are the changes in the alpha- and beta-band PLV between pedal stages. The paired permutation test is used to compare the distributions of PLV changes between low- and high-resistance exercises, where the mark * indicates a significance level of *p* < 0.05. The effect size is reported as the correlation coefficient ρ based on the Wilcoxon signed-rank test

**Fig. 7:**
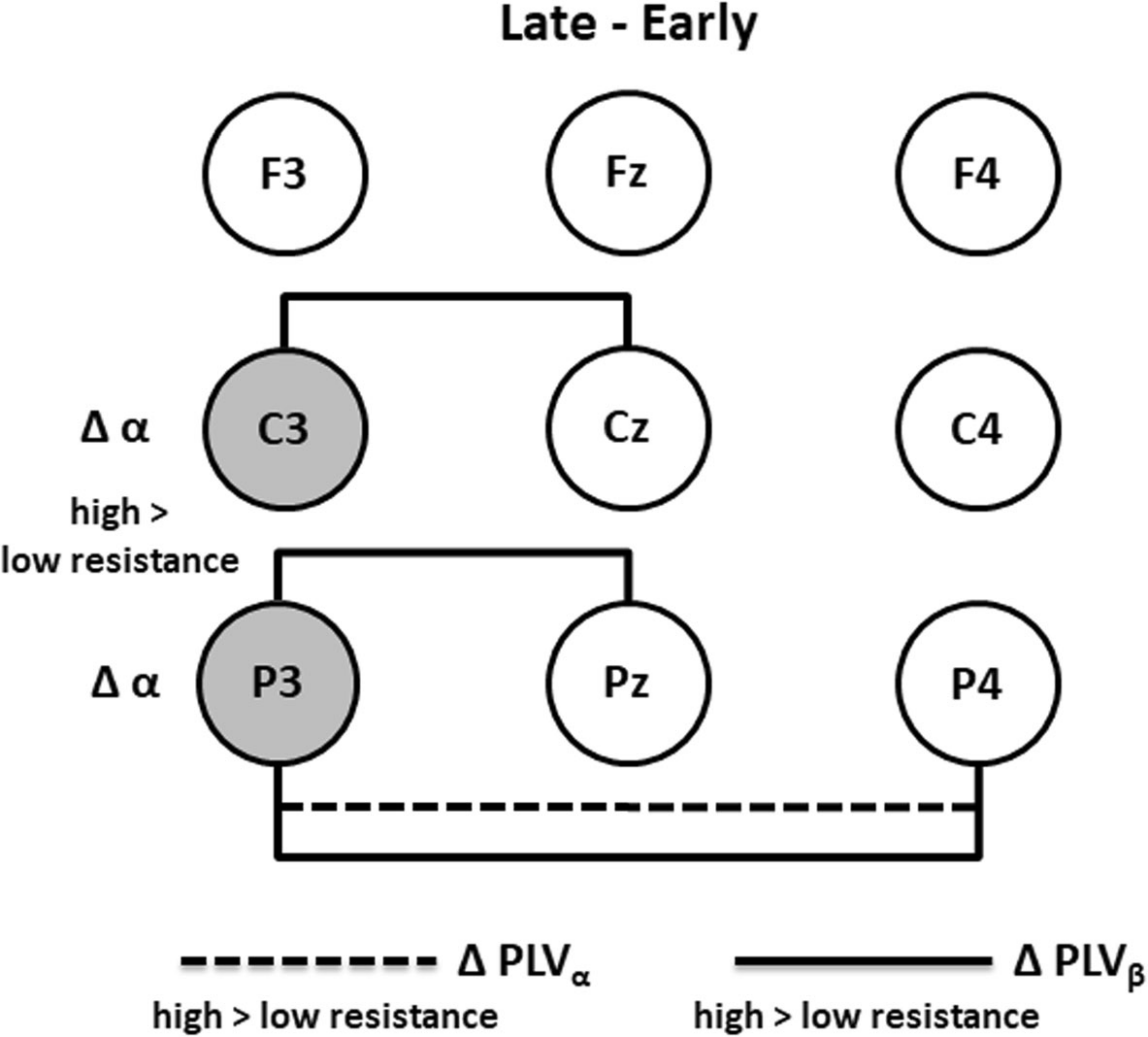
The topology of significant phase-locking value changes in the alpha band (ΔPLV_α_) and in the beta band (ΔPLV_β_) as well as significant change rate in the alpha-band power (Δα) measured in the transition from the early to the late pedal stage between low- and high-resistance exercise

Figure 7 summarizes the changes of brain activities from the early to the late pedal stage across exercise resistances by the topology of inter-cerebral links and electrodes with statistically significant differences. High-resistance exercise produced significantly higher beta-band PLV changes in left central (C3-Cz) and left parietal (P3-Pz) links, and significant higher alpha- and beta-band PLV changes in inter-parietal (P3-P4) link. In addition to these significant links, significant higher change rate in alpha-band power was exhibited in the left central and left parietal areas (C3 and P3).

## Discussion

The Borg RPE and mean heart rate increased from early to middle to late pedal stages, demonstrating that both of the exercise regimes used in this study produce a sustained increase in feeling of fatigue and activation of the sympathetic input [39, 40] over the duration of the exercise. This finding is consistent with general exercise physiology, and confirms that the two physiological metrics can be used as independent variables modulating brain activity, proxied in this study by the three pedal stages.

In this study, all subjects reported a maximum value of Borg RPE during high-resistance cycling exercise, indicating that they felt extremely hard, therefore the exercise was stopped. Nevertheless, only 3 subjects reported a maximum value of Borg RPE during low-resistance cycling exercise. In addition, the Borg RPE is a subjective estimate of exercise effort whereas inter-individual variability in Borg RPE was observed at both relative exercise intensities of 60 and 80% of maximal heart rate reserve [43]. Therefore, we considered time as an additional factor of fatigue. The exercise period is then divided into early, middle and late stages.

The exercise-state EEG provides a convenient approach to study brain activity in response to motor tasks. It is complementary to the connectivity information derived from functional magnetic imagery or near-infrared ray spectroscopy. Several studies have observed the formation of functional associations during sustained constant-intensity exercise [23] or incremental graded-intensity cycling exercise [24, 25] and the reallocation of cerebral resources caused by additional audio stimuli during walking or cycling [44, 45]. These studies demonstrate that the brain connectivity is modulated as muscle fatigue or exercise intensity increases. Nevertheless, the effect of different exercise intensity on brain connectivity, not based on the increased exercise intensity has not been reported whereas it is demonstrated by the separate-day experimental design in our study.

The separate-day experimental setup has been used before to compare alpha-band activations between a controlled-pace incremental exercise and a self-paced 4-km cycling exercise [46]. In contrast, our study investigates the effect of exercise intensity on several types of brain activity, for two types of cycling exercise. Applying different exercises on separate days has the advantage of starting each experimental measurement under fresh conditions, to avoid the influence of a previous exercise session. However, the subject’s baseline between separate-day measurements can also vary, which may affect the derived parameters. Therefore, we use the changes in EEG powers and phase-locking values between the early, middle, and late pedal stages to investigate the effect of exercise intensity on brain activity.

We observed significant change rate in alpha-band power and significant change in phase-locking values from the early to the late pedal stages, but this effect appeared mainly in the high-resistance exercise. The high-resistance pedal exertion might strengthen the inter-cerebral connections, which take on more workload during the fatigue process. Specifically, the elevated left central and left parietal beta-band connectivity and inter-parietal alpha- and beta-band connectivity may be attributed to the need to integrate sensory information from the periphery and enhance the communication with the motor cortex during fatigue [24].

In this study, both low- and high-resistance exercises produced gradual increases in the Borg RPE and mean heart rate from early to middle to late pedal stages, but only high-resistance pedaling reported the increased EEG power in alpha and beta bands, in particular in frontal and central areas. In previous ergometer cycling studies, incremental graded exercises were observed to cause increased α power in the central and parietal areas [15] and increased EEG current density in the primary motor area [14]. Intensified EEG in the frontal cortex is also used as an indication of fatigue during high-cadence, fixed-resistance cycling exercise [16]. All subjects in our study actually reported maximal Borg RPE in late pedal stage in high-resistance exercise, indicating that they felt extremely hard. This fatigue situation was also accompanied by the increases in α and β power as the findings in previous researches.

On the other hand, different resistance pedaling produced individual PLV alterations along the pedal stages. A significant increase in the beta-band PLV was observed in left central area in high-resistance, late-stage pedaling; however, the PLV in the parietal area decreased from early to middle and from early to late stage in low-resistance exercise. As well as the finding in a previous study, the incremental graded-intensity cycling exercise resulted in increased phase synchronization in the period of physical fatigue [24], we infer that the enhanced cortico-cortical coupling is a necessary mechanism of physical fatigue induced by high-resistance exercise. In contrast, the various-type task produces different alteration in brain connectivity in the period of mental fatigue: EEG phase synchronization increased as the mental state of drivers shifts from alertness to fatigue [18]; EEG coherences were slightly increased at the end of the 1-h monotonous driving session [19]; A long-term cognitive task induced a significant decrease in EEG coherences and PLVs [47]; Brain connective network was reorganized after 90-min simulated driving-induced mental fatigue [22]. In our study, the late-stage decrease in PLV in low-resistance exercise may be linked with less demand of cerebral associations to perform low physical-effort task unlike the high effort-demanding in high-resistance pedaling.

The EEG in higher frequencies (> 20 Hz) overlaps with the spectral width of muscle activity [27, 48]. Therefore, EEG coherence and phase synchronization as well as EEG power in alpha band only [24] or in conjunction with beta band [10, 15, 23, 25, 44, 49] are commonly used to study brain activation and connectivity during exercise. In this study, we focus on brain activity in alpha band (8–13 Hz) and lower beta band (14–20 Hz) to reduce potential risks of electromyogram contamination. In addition, independent vector analysis is used to remove the most likely muscle-related component. The exclusion of the abruptly changed 1-s segments from analysis further reduce the effect of muscle artifacts on the estimation of EEG power and phase-locking values.

Some exercise-related EEG studies showed the expected results based on a limited number of participants [9, 10, 16, 49, 50]. Although this study is also based on a limited number of participants, significant differences have been observed in several variables between different pedal stages or between low- and high-resistance exercises, which could support a larger population study in the future. We also used a nonparametric comparison to reduce the effect of the possible un-normality in some variables caused by the limited number. In addition, the effect size (i.e., rank correlation of the two distributions) of the test quantifies the statistical difference between a set of paired measurements. Since the subjects have different physiological baselines and responses; nonetheless we expect their brain activity to change in similar ways. Their individual differences can be compensated by assuming that the rank ordering of the subjects remains similar when comparing two sets of measurements, without relying overmuch on the specific values of the individual measurements. Accordingly, a medium to high effect size is demonstrated in the variables which are statistically different between different pedal stages and in the variables which are statistically different between low- and high-resistance exercises.

## Conclusion

EEG spectral powers and phase-locking values are used to investigate the effect of exercise intensity on brain activity, under a separate-day experimental configuration. To the best of our knowledge, this is the first report to investigate the effect of pedal resistance on EEG phase-locking values. Our findings support the mechanism of cerebral adaption induced by high-intensity but not low-intensity exercise, specifically the recruitment of additional brain connectivity as well as cortical activations.

## Data Availability

The datasets generated during and/or analyzed during the current study are available from the corresponding authors on reasonable request.

## Abbreviations

EEG: Electroencephalogram
RPM: Revolutions per minute
RPE: Rating of perceived exertion
PLV_α_: Alpha-band phase-locking value
PLV_β_: Beta-band phase-locking value
